# Relationship between Vitamin D Status and Autonomic Nervous System Activity

**DOI:** 10.3390/nu8090565

**Published:** 2016-09-13

**Authors:** Morton G. Burt, Brenda L. Mangelsdorf, Stephen N. Stranks, Arduino A. Mangoni

**Affiliations:** 1 Southern Adelaide Diabetes and Endocrine Services, Repatriation General Hospital, Adelaide, SA 5041, Australia; Brenda.Mangelsdorf@sa.gov.au (B.L.M.); Steve.Stranks@sa.gov.au (S.N.S.); 2 School of Medicine, Flinders University, Adelaide, SA 5042, Australia; Arduino.Mangoni@sa.gov.au; 3 Department of Clinical Pharmacology, Flinders Medical Centre, Adelaide, SA 5042, Australia

**Keywords:** 25 hydroxy vitamin D, pulse wave velocity, augmentation index, baroreflex sensitivity, metanephrine

## Abstract

Vitamin D deficiency is associated with increased arterial stiffness. However, the mechanisms underlying this association have not been clarified. The aim was to investigate whether changes in autonomic nervous system activity could underlie an association between 25 hydroxy vitamin D and arterial stiffness. A total of 49 subjects (age = 60 ± 8 years, body mass index = 26.7 ± 4.6 kg/m^2^, 25 hydroxy vitamin D = 69 ± 22 nmol/L) underwent measurements of pulse wave velocity (PWV) and augmentation index (AIx), spontaneous baroreflex sensitivity, plasma metanephrines and 25 hydroxy vitamin D. Subjects with 25 hydroxy vitamin D ≤ 50 nmol/L were restudied after 200,000 International Units 25 hydroxy vitamin D. Plasma metanephrine was positively associated with AIx (*p* = 0.02) independent of age, sex, smoking and cholesterol and negatively associated with 25 hydroxy vitamin D (*p* = 0.002) independent of age, sex and season. In contrast, there was no association between baroreflex sensitivity and 25 hydroxy vitamin D (*p* = 0.54). Treatment with vitamin D increased 25 hydroxy vitamin D from 43 ± 5 to 96 ± 24 nmol/L (*p* < 0.0001) but there was no significant change in plasma metanephrine (115 ± 25 vs. 99 ± 39 pmol/L, *p* = 0.12). We conclude that as plasma metanephrine was negatively associated with 25 hydroxy vitamin D and positively with AIx, it could mediate an association between these two variables. This hypothesis should be tested in larger interventional studies.

## 1. Introduction

Vitamin D is a steroid hormone that has clinical effects beyond its established role in skeletal health. Low concentrations of 25-hydroxy vitamin D and 1,25-dihydroxy vitamin D are associated with increased cardiovascular events and mortality in observational studies [1,2,3]. However, it is unclear whether vitamin D deficiency increases cardiovascular mortality directly or is a biomarker for another medical condition or pathophysiological pathway that alters cardiovascular risk. There are no sufficiently powered interventional studies to determine an effect of vitamin D treatment on cardiovascular events. Lacking this data, investigation of mechanisms by which vitamin D deficiency could affect cardiovascular physiology may aid interpretation of the relationship between vitamin D and cardiovascular health.

Increased arterial stiffness and arterial wave reflection, an indirect marker of arterial stiffness, have been proposed as mechanisms linking vitamin D deficiency and cardiovascular events. These measures, considered major determinants of systolic blood pressure and pulse pressure, and consequently cardiovascular risk [4,5], have been negatively associated with vitamin D concentration [6,7,8]. Arterial stiffness is increased by arterial wall pathology such as atherosclerosis and calcification and is also tonically regulated by endothelial nitric oxide production [9]. Vitamin D deficiency has been associated with these structural and functional vascular disorders in some studies [6,10,11], but not in others [12]. Therefore, further investigation of mechanisms linking serum vitamin D and arterial stiffness is warranted.

The autonomic nervous system is another major regulator of arterial stiffness [13] and autonomic dysfunction is associated with an increased risk of cardiovascular mortality [14]. However, few studies have investigated the relationship between vitamin D and autonomic nervous system activity. Low concentrations of 1,25 dihydroxy vitamin D, but not 25 hydroxy vitamin D, were associated with autonomic dysfunction in response to angiotensin II challenge [15]. However, 25 hydroxy vitamin D treatment attenuated an unfavourable autonomic nervous system response to angiotensin II [16]. In contrast, an in vitro study reported 1,25-dihydroxyvitamin D increased tyrosine hydroxylase mRNA, suggesting that vitamin D might stimulate the rate limiting step in catecholamine biosynthesis [17]. With these conflicting results, there is interest in further investigation into the relationship between vitamin D and autonomic nervous system activity [18].

Increased renin secretion has also been proposed as a mechanism linking vitamin D deficiency and cardiovascular events. In animal models, vitamin D deficiency has been associated with increased renin secretion [19]. As the sympathetic nervous system stimulates renin secretion, increased sympathetic nervous system activity could mediate an association between vitamin D deficiency and renin secretion.

The aim of this study was to investigate whether changes in autonomic nervous system activity could underlie an association between serum vitamin D and arterial stiffness. We hypothesized that autonomic dysfunction could mediate an association between vitamin D and arterial stiffness, either directly or indirectly via an effect on the renin-angiotensin-aldosterone system.

## 2. Methods

### 2.1. Subjects

We recruited 49 subjects aged 30–80 years both from the Metabolic Bone Clinic, Repatriation General Hospital and from the general community by advertisement in local newspapers. Subjects were excluded if they had taken calcium or vitamin D supplements in the prior 3 months or if they were taking a beta blocker medication, had diabetes mellitus, atrial fibrillation or Raynaud’s phenomenon, were pregnant or were unable to provide written informed consent. Two subjects were excluded from the statistical analyses; one because their blood results revealed previously undiagnosed hypoparathyroidism and one because limited venous access precluded measurement of serum vitamin D. Therefore, 47 subjects were included in the final analysis of most variables. Only 43 subjects had sufficient plasma stored for metanephrine analysis.

### 2.2. Study Design

These studies were undertaken within the Endocrine Research Unit, Repatriation General Hospital, Adelaide, Australia. The study was approved by the Research and Ethics Committee, Repatriation General Hospital (Number 42/07, approved 27 July 2009) and all subjects provided written informed consent. Subjects attended the Endocrine Research Unit at 08.30 a.m. after an overnight fast. After resting for 30 min, pulse wave analysis, pulse wave velocity (PWV) and an assessment of autonomic nervous system activity were performed and then a blood sample was collected. Subjects with a serum 25 hydroxy vitamin D concentration ≤50 nmol/L were restudied 4–6 weeks after administration of two oral doses of 100,000 International Units 25 hydroxy vitamin D administered two weeks apart (Biological Therapies, South Australia, Australia).

### 2.3. Pulse Wave Analysis

Pulse wave analysis was performed using a SphygmoCor device (AtCor Medical, West Ryde, NSW, Australia) and a high-fidelity micromanometer (SPC-301, Millar Instruments, Houston, TX, USA) as previously described [20,21]. One investigator (BLM) performed all measurements. The radial pulse waveform was recorded and central aortic pressure derived using an automated generalized transfer function. The augmentation index (AIx) was calculated as the increment in pressure from the first shoulder in the ascending aortic pressure wave to the peak of this wave. The day-to-day intraclass correlation for AIx for this operator is 0.82. To correct for differences in pulse rate, AIx results were normalized to a heart rate of 75 beats/min. The quality control indices within the SphygmoCor device software were used to verify the quality of recorded waveforms. If indices were outside acceptable limits the waveform was discarded and the measurement repeated. Results for AIx are at an average of five consecutive recordings.

### 2.4. Pulse Wave Velocity

PWV was performed by combining pulse wave analysis at the carotid and femoral arteries with simultaneous electrocardiograph recording [20]. PWV is calculated by measuring the difference in time the pulse waveform takes to reach the carotid and femoral arteries. Reported results are the average of six consecutive recordings. The average day-to-day coefficient of variation (CV) for PWV is 7.6%.

### 2.5. Autonomic Nervous System Activity

Plasma normetanephrine and metanephrine were measured after solid-phase extraction from plasma using a5500 tandem mass spectrometer (AB Sciex Australia Pty Ltd., Mount Waverley, VIC, Australia) [22]. The between-run CV of the method is <6% for normetanephrine and metanephrine. Spontaneous baroreflex sensitivity quantified using the sequence method and heart rate spectral parameters were measured using a Taskforce Haemodynamic Monitor 3040i (CNSystems, Graz, Austria) [23]. Four electrodes were placed on the subject’s anterior chest for measurement of heart rate and a “flying V” finger cuff on the subject’s hand to measure beat-by-beat arterial pressure, allowing simultaneous analysis of heart rate and blood pressure variability. Following resting supine for 10 min, recordings were performed for 20 min.

### 2.6. Other Laboratory Analysis

25 hydroxy vitamin D and 1,25 dihydroxy vitamin D were measured by chemiluminescent assays (IDS-iSYS, IDS Ltd., Boldon, UK) with CVs of 7%–11% and 10%–18% respectively. Renin and aldosterone were measured by chemiluminescent assays (Diasorin, Stillwater, MN, USA) with CVs of <10% and <5% respectively. PTH was measured by electrochemiluminescent immunoassay (Roche Diagnostics GmbH, Mannheim, Germany) with CV of <4%. Fasting lipid profiles were measured by enzymatic colorimetry (Roche Modular P Unit; Roche Diagnostics GmbH, Mannheim, Germany).

### 2.7. Statistical Analysis

Data were analysed using SPSS 20.0 for Windows (SPSS Inc., Chicago, IL, USA) with *p* < 0.05 considered statistically significant. Results are presented as mean ± standard deviation if normally distributed and median (interquartile range) if the distribution was not normal. First, multivariable linear regression analyses were performed to assess the relationship between measures of autonomic nervous system activity and arterial stiffness with age, sex, smoking status and serum cholesterol covariates in these analyses. Variables that were not normally distributed were log-transformed prior to these analyses to achieve a normal distribution. Next, multivariable linear regression analyses assessed the relationship between measures of autonomic nervous system activity and 25 hydroxy vitamin D, 1,25 dihydroxy vitamin D and PTH with age, sex, and season covariates in these analyses. A subgroup analysis was then performed on the 37 subjects who were not taking anti-hypertensive therapy to assess whether measures of autonomic nervous system activity that correlated with vitamin D were correlated with serum renin or aldosterone. Finally, changes in variables after vitamin D treatment were assessed using paired *t*-tests for normally distributed data and a Wilcoxon Signed Rank test for data where the distribution was not normal. The primary endpoints were the relationship between 25 hydroxy vitamin D and PWV and baroreflex sensitivity.

## 3. Results

### 3.1. Subject Characteristics

Thirty-five of the 47 subjects were female. The subjects’ mean age was 60 ± 8 years, weight was 74 ± 15 kg, height was 1.66 ± 0.09 m, body mass index was 26.7 ± 4.6 kg/m^2^ and waist circumference was 89 ± 13 cm. Only one subject had a history of cardiovascular or cerebrovascular disease, five subjects were current smokers and 10 subjects were treated for hypertension. The subjects’ 25 hydroxy vitamin D was 69 ± 22 nmol/L, 1,25 dihydroxy vitamin D was 102 (85–129) pmol/L, normetanephrine was 340 (260–450) pmol/L and metanephrine was 98 ± 29 pmol/L.

### 3.2. Associations between Arterial Stiffness and Autonomic Nervous System Activity

Baroreflex sensitivity and high frequency heart rate variability were negatively associated with PWV, independent of age, sex, smoking status and serum cholesterol (Table 1). Low frequency heart rate variability, LF:HF ratio, plasma normetanephrine and plasma metanephrine were not independently associated with PWV. Plasma metanephrine was positively associated with AIx, independent of age, sex, smoking status and serum cholesterol (Table 1). Baroreflex sensitivity, low frequency heart rate variability, high frequency heart rate variability, LF:HF ratio and plasma normetanephrine were not independently associated with AIx.

### 3.3. Associations between Autonomic Nervous System Activity and Vitamin D/PTH

**Figure 1:**
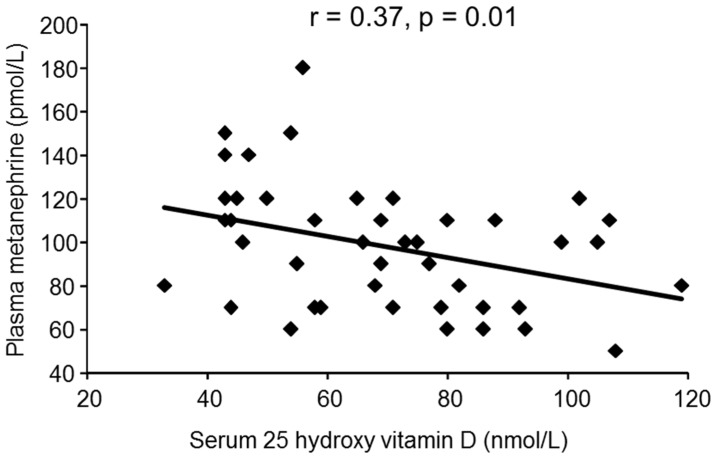
Simple linear regression relationship between serum 25 hydroxy vitamin D and plasma metanephrine in 43 subjects.

Figure 1 depicts the univariable relationship between serum 25 hydroxy vitamin D concentration and plasma metanephrine. Plasma metanephrine was negatively associated with serum 25 hydroxy vitamin D concentration, independent of age, sex and season (Table 2). Baroreflex sensitivity, low frequency heart rate variability, high frequency heart rate variability, LF:HF ratio and plasma normetanephrine were not independently associated with serum 25 hydroxy vitamin D concentration (Table 2). The negative correlation between 25 hydroxy vitamin D and AIx, albeit similar to that reported in previous studies [6], was not statistically significant (*r* = −0.24, *p* = 0.10). 1,25 dihydroxy vitamin D and PTH were not significantly associated with measures of autonomic nervous system activity (Table 2).

### 3.4. Associations with the Renin-Angiotensin-Aldosterone System

In the 37 subjects who were not taking anti-hypertensive therapy, there was no correlation between plasma metanephrine concentration and renin (*r* = 0.08, *p* = 0.68) or aldosterone (*r* = 0.04, *p* = 0.83). There were no significant correlations between 25 hydroxy vitamin D concentration and renin (*r* = 0.10, *p* = 0.56) or aldosterone (*r* = 0.04, *p* = 0.84). There were no significant correlations between 1,25 dihydroxy vitamin D concentration and renin (*r* = 0.10, *p* = 0.57) or aldosterone (*r* = 0.12, *p* = 0.52).

### 3.5. Effect of Vitamin D Replacement

This analysis includes the 12 subjects with 25 hydroxy vitamin D <50 nmol/L. Vitamin D treatment increased mean 25 hydroxy vitamin D by 53 nmol/L and 1,25 dihydroxy vitamin D by 33 pmol/L (Table 3). The increase in pulse rate after vitamin D treatment almost reached statistical significance (*p* = 0.053). There were no significant changes in renin and aldosterone, blood pressure, PWV, AIx, baroreflex sensitivity, heart rate spectral parameters and plasma metanephrine and normetanephrine following vitamin D treatment (Table 3).

## 4. Discussion

In this study we have investigated whether changes in autonomic nervous system activity could underlie an association between serum vitamin D and arterial stiffness. There was an independent association between several measures of autonomic nervous system activity and arterial stiffness. However, only plasma metanephrine was associated with 25 hydroxy vitamin D. This suggests that vitamin D could modulate catecholamine synthesis and/or secretion from the adrenal medulla, which could contribute to the relationship between serum vitamin D and cardiovascular risk.

We found that different measures of autonomic nervous system activity were associated with PWV and AIx. There are differences in the physiologic principles underlying measures of both arterial stiffness and autonomic nervous system activity that are likely to explain any discordance. Carotid-femoral PWV is considered the gold standard measure of arterial stiffness as it directly reflects arterial stiffness in the aorta [24]. However, AIx is an indirect marker of arterial stiffness that is also influenced by other variables, in particular the pattern and duration of left ventricular ejection [24]. Quantification of baroreflex sensitivity by the sequence method and heart rate variability by spectral analysis predominantly reflect parasympathetic nervous system activity [25]. Plasma normetanephrine is produced in both the sympathetic nervous system and adrenal medulla [26]. In contrast, plasma metanephrine is mainly a measure of catecholamine synthesis in the adrenal medulla as over 90% of circulating plasma metanephrine is produced in the adrenal glands [26].

PVW was independently associated with baroreflex sensitivity and high frequency heart rate variability. As these are both measures of parasympathetic nervous system activity, our data suggest that the parasympathetic nervous system is an important regulator of arterial stiffness. AIx was not associated with baroreflex sensitivity or heart rate spectral parameters, but was independently associated with plasma metanephrine. The lack of association between PWV and plasma metanephrine suggests that the latter does not influence arterial stiffness per se. Therefore, the association between AIx and plasma metanephrine could be explained by adrenaline and its metabolites stimulating cardiac output, which would affect AIx, but not PWV.

In this study, 25 hydroxy vitamin D was negatively correlated with plasma metanephrine (Figure 1). Furthermore, the association was stronger when the relationship was corrected for age, sex and season. As plasma metanephrine is mainly derived from the adrenal glands [26], we propose that low concentrations of 25 hydroxy vitamin D could stimulate catecholamine production in the adrenal medulla. It has been postulated that vitamin D could cross the blood brain barrier and modulate autonomic nervous system activity centrally [18]. However, while we cannot exclude an effect of vitamin D on central nervous system activity, our study suggests that vitamin D status is predominantly associated with adrenal medullary function.

Plasma noradrenaline and adrenaline concentrations are higher in winter than in summer [27]. Plasma normetanephrine follows a similar seasonal pattern [28]. The seasonal variability in plasma metanephrine concentration is less marked, but metanephrine concentrations are higher in spring than in autumn [28]. Stimulation of the sympathetic nervous system by cold temperature has been proposed as a mechanism to explain these findings. However, the negative association between 25 hydroxy vitamin D and plasma metanephrine raises the possibility that low vitamin D concentrations could contribute to seasonal changes in adrenaline and metanephrine. The lack of association between 25 hydroxy vitamin D and normetanephrine suggests vitamin D status is unlikely to underlie seasonal variation in plasma normetanephrine.

We recognize that an association between 25 hydroxy vitamin D and plasma metanephrine does not demonstrate cause and effect. Although our subjects did not have an obvious intercurrent illness, a low 25 hydroxy vitamin D could be a surrogate for another variable that stimulates catecholamine secretion. In our interventional study, plasma metanephrine fell by 14% after vitamin D supplementation, however this change was not statistically significant. While this could represent a type 2 error, there was an increase in heart rate following vitamin D replacement that approached statistical significance and no change in systolic or diastolic blood pressure. Furthermore, a meta-analysis reported no effect of vitamin D supplementation on systolic or diastolic blood pressure [29]. These findings are not consistent with the hypothesis that replenishing vitamin D stores reduces catecholamine secretion from the adrenal medulla. However, our interventional study was small and the effect of vitamin D on blood pressure could be multifactorial. Our cross-sectional study suggests a need for larger studies investigating the effect of vitamin D replacement on adrenal medullary function.

25 hydroxy vitamin D was not significantly associated with baroreflex sensitivity or heart rate spectral parameters, suggesting it does not alter basal parasympathetic nervous system activity. A previous study reported that vitamin D does affect parasympathetic nervous system activity [16]. However, in that study, the effect of vitamin D on parasympathetic nervous system activity became apparent during an angiotensin II challenge [16]. While we may have missed this effect, we did investigate subjects under conditions they will experience during their day-to-day lives.

There were no significant associations between 1,25 dihydroxy vitamin D or PTH and measures of autonomic nervous system activity. 1,25 dihydroxy vitamin D was reported to stimulate tyrosine hydroxylase activity in vitro [17]. In contrast, we did not find a significant association between 1,25 dihydroxy vitamin D and plasma metanephrine in vivo. Previous studies have suggested that PTH, which is affected by vitamin D status, may directly regulate arterial stiffness [30]. However, there were no significant associations between PTH and measures of autonomic nervous system activity, suggesting that any effect of PTH on arterial stiffness is not mediated by the autonomic nervous system.

Stimulation of the renin-angiotensin-aldosterone system has been proposed as a mechanism linking vitamin D deficiency and cardiovascular disease [19]. As the sympathetic nervous system stimulates renin secretion, we hypothesized that this relationship might be indirect and mediated by changes in autonomic nervous system activity. However, there was no correlation between plasma metanephrine—the only measure of autonomic nervous system activity associated with 25 hydroxy vitamin D concentration—and renin or aldosterone. As such, we did not find evidence that a relationship between vitamin D deficiency and the renin-angiotensin-aldosterone system is modulated by the autonomic nervous system.

We acknowledge that our study has limitations. Firstly, the cross-sectional study may show an association but does not establish cause and effect. However, it does provide insight into appropriate endpoints for future interventional studies, in particular adrenal catecholamine secretion. Secondly, the sample size was relatively small. Nevertheless, the cross-sectional study sample size was sufficient to show relationships between autonomic nervous system activity and arterial stiffness that were independent of other variables. Thirdly, there are more refined techniques to measure sympathetic nervous system activity available than plasma metanephrines, which additionally have a shorter half-life than that of 25 hydroxy vitamin D. However, plasma samples were collected after fasting and supine, conditions shown to reduce variability in plasma metanephrines [31]. Fourthly, we did not measure serum oestradiol, and variability throughout the menstrual cycle could potentially influence vascular function. Nevertheless, this is unlikely to have had a major effect on results as only two subjects were female and aged ≤50 years. Finally, we did not measure fat mass, which may have influenced 25 hydroxy vitamin D concentration.

In summary, baroreflex sensitivity, high frequency heart rate variability and plasma metanephrine were associated with measures of arterial stiffness. However, only plasma metanephrine was significantly associated with 25 hydroxy vitamin D. This suggests that plasma metanephrine could mediate an association between 25 hydroxy vitamin D and AIx. Future larger interventional studies should investigate whether vitamin D treatment reduces catecholamine secretion from the adrenal medulla and whether this reduces arterial stiffness and other surrogate markers of cardiovascular risk.

## Figures and Tables

**Table 1:** Multiple regression analyses of the relationship between measures of autonomic nervous system activity and arterial stiffness in 47 subjects.

	Pulse Wave Velocity *		Augmentation Index	
	β Coefficient	*p* Value	β Coefficient	*p* Value
**Heart rate variability**				
Baroreflex sensitivity *	**−0.30**	**0.04**	+0.01	0.93
Low Frequency (LF) band *	−0.26	0.06	+0.10	0.44
High Frequency (HF) band *	**−0.33**	**0.02**	+0.10	0.47
LF:HF *	+0.04	0.14	+0.05	0.72
**Plasma catecholamine metabolites**				
Normetanephrine *^,#^	+0.09	0.97	+0.10	0.48
Metanephrine ^#^	+0.00	0.46	**+0.34**	**0.02**

* Variable log-transformed before multiple regression analysis; ^#^ 43 subjects included in analysis; *p* values are independent of age, sex, smoking status and serum cholesterol.

**Table 2:** Multiple regression analyses of the relationship between vitamin D/parathyroid hormone (PTH) and measures of autonomic nervous system activity in 47 subjects.

	25 Hydroxy Vitamin D		1,25 Dihydroxy Vitamin D		PTH	
	β Coefficient	*p* Value	β Coefficient	*p* Value	β Coefficient	*p* Value
**Heart rate variability**						
Baroreflex sensitivity *	−0.10	0.54	−0.25	0.10	−0.03	0.85
Low Frequency (LF) band *	+0.03	0.87	−0.30	0.07	−0.12	0.48
High Frequency (HF) band *	+0.07	0.65	−0.24	0.15	−0.03	0.86
LF:HF *	+0.00	0.99	+0.05	0.76	−0.15	0.35
**Plasma catecholamine metabolites**						
Normetanephrine *^,#^	−0.02	0.91	−0.08	0.65	−0.08	0.64
Metanephrine ^#^	**−0.55**	**0.002**	−0.04	0.83	+0.21	0.28

* Variable log-transformed before multiple regression analysis. PTH = parathyroid hormone; ^#^ 43 subjects included in analysis; *p* values are independent of age, sex and season.

**Table 3:** Effect of the administration of 200,000 International Units vitamin D on the renin angiotensin system and cardiovascular function in 12 subjects.

	Before	After	*p* Value
25 hydroxy vitamin D (nmol/L)	**43** **± 5**	**96** **± 24**	**<0.0001**
1,25 dihydroxy vitamin D (pmol/L)	**113** **± 39**	**146** **± 67**	**0.02**
Renin (μIU/mL)	7.5 (3.1–15.5)	8.1 (4.4–12.7)	0.44
Aldosterone (pmol/L)	133 ± 89	188 ± 198	0.15
Pulse (beats/min)	65 ± 6	68 ± 10	0.053
Systolic BP (mmHg)	132 ± 22	131 ± 18	0.91
Diastolic BP (mmHg)	76 ± 10	76 ± 10	0.91
Pulse wave velocity (m/s)	8.3 (6.6–9.3)	8.6 (6.5–10.5)	0.37
Augmentation index (%)	28 ± 5	26 ± 7	0.21
Baroreceptor sensitivity (m/s)	13.6 (8.8–17.0)	14.9 (8.8–18.9)	0.64
Low Frequency band	202 (112–661)	297 (101–517)	0.64
High Frequency band	216 (95–634)	301 (152–786)	0.29
LF:HF	0.8 (0.6–1.0)	0.8 (0.6–1.0)	0.72
Normetanephrine (pmol/L)	300 (260–530)	300 (200–390)	0.45
Metanephrine (pmol/L)	115 ± 25	99 ± 39	0.12

Values represent mean ± standard deviation or median (interquartile range). BP = blood pressure.
